# A Microstructural Investigation of Austenitic Heat Resistant Alloy after 500 h of Steam Oxidation

**DOI:** 10.3390/ma14061453

**Published:** 2021-03-16

**Authors:** Bogdan Rutkowski, Krzysztof Baran, Remigiusz Błoniarz, Tomasz Kozieł

**Affiliations:** Faculty of Metals Engineering and Industrial Computer Science, AGH University of Science and Technology, Al. A. Mickiewicza 30, 30-059 Kraków, Poland; kris@agh.edu.pl (K.B.); bloniarz@agh.edu.pl (R.B.); tkoziel@agh.edu.pl (T.K.)

**Keywords:** steam oxidation, TEM, SEM, austenitic steel

## Abstract

Alloy 709 was oxidized at 700 °C for 500 h in a steam environment. A microstructural analysis of the oxide scale is reported. Modern techniques of advanced electron microscopy were used to characterize the morphology of the oxide scale and recognize its single components. The material developed a complex, multilayered oxide scale. The outermost layer consisting of Fe_2_O_3_. Fe_2_NiO_4_ tI28 spinel was detected underneath. An internal oxidation zone is present in the innermost layer. High quality SEM-EDS maps give insight into a larger area of the oxide scale at a relatively low magnification.

## 1. Introduction

A lot of effort has been made in order to improve Fe-based alloys to increase their operation temperature in power plants, which would allow significant savings of hard coal or lignite in energy production. It would also induce a decrease in CO_2_ emission to the atmosphere. Until now, the 9–12% Cr martensitic steels have been very widely used. Among others, they are relatively inexpensive due to the lack of Ni and have a smaller thermal expansion coefficient, which is important in running up and shutting down cycles. Unfortunately, these steels can operate below 620 °C [1,2], which is insufficient in modern power generation systems. Creep strength is strongly decreased above 620 °C [3,4], and corrosion resistance is smaller due to a relatively low amount of Cr [5]. Therefore, continuous work is performed to develop new Ni-based alloys and steels capable of operation at around 700 °C. The first of the abovementioned alloys are able to operate at such a temperature; however, their usage is not economical due to the high prices of Ni. Austenitic steels can fill the gap between martensitic steels and Ni-based alloys. First, Cr content has to be raised to around 22%, which will allow for the formation of a continuous, passive layer of Cr_2_O_3_ at the surface [6]. Since Cr stabilizes ferrite structure, its addition has to be compensated by an element which has a strong impact on austenite stabilization (usually Ni). According to Schaeffler’s diagram [7], above 16% of Ni has to be added to stabilize the austenitic structure of steel with 22% Cr. Theoretically, this is enough to maintain good corrosion resistance. In addition, there is also creep resistance. This is assured by the blocking of dislocation movement [4,8]. Such obstacles can be atomic lattice distortions caused by the presence of alloying element atoms with the different atomic radii in comparison to the matrix atoms. The addition of W induces such an effect [9]. Moreover, the addition of other alloying elements (eg. Ti, Nb, N) will cause precipitation of very fine primary or secondary phases, which will take part in the precipitation strengthening process [10]. Such additions, however, may induce complications in the corrosion processes, making them even more complex.

Alloy 709, due to its reasonable chemical composition is used in power plants. It is foreseen as a good candidate to work at temperatures higher than those possible for martensitic steels. Its creep properties as well as microstructure development during ageing were studied elsewhere [11,12,13,14,15,16,17]; however, only little information about oxidation behavior exists [18]. In the current paper, the oxidation behavior of modern, Fe–20Cr–25Ni-based steel is reported. The prepared samples were oxidized in steam at 700 °C up to 500 h and further investigated by mains of advanced scanning and transmission electron microscopy techniques which allow one to obtain knowledge about behavior of the steel.

## 2. Materials and Methods

The exact chemical composition of the investigated material is given in Table 1. It was synthesized from Fe Armco, pig Fe (Fe-4.3 wt.% C), 316L steel and high purity elemental Cr, Nb, Ni, Ti metals. This mixture was arc melted under a Ti-gettered argon atmosphere using Arc Melter AM (Edmund Bühler GmbH, Bodelshausen, Germany) on a water-cooled copper hearth. The alloy was remelted five times in order to achieve chemical homogeneity.

The as-cast laboratory ingot with the weight of 100 g and initial thickness of ca. 10 mm was heated and homogenized at 1200 °C (1473 K) for 40 min. Further rolling was carried out with the use of the 4-high laboratory rolling mill, with a working rolls diameter of 100 mm. The rotational speed was 80 rev/min. A thermomechanical scheme was maintained to ensure dynamic recrystallization conditions during deformation. This was achieved through additional heating (a 10 min period at 1200 °C) every 2 passes. The thickness was reduced by 1 mm for each pass, except for the last two passes, then the thickness reduction was 0.5 mm per pass. A thickness of 3 mm was finally obtained. The as-rolled specimens were reheated for 5 min at 1200 °C in order to annihilate the microstructural effects of the deformation and then cooled in the air. The obtained material has a grain size of 51.7 (±16.7) μm. Afterwards, samples for oxidation experiments in the form of 12 mm × 10 mm × 2 mm coupons were prepared. Each coupon was carefully ground on abrasive papers up to 4000 grit and polished with diamond suspensions (with the final size being 0.25 μm). Each coupon had a hole drilled to mount it properly in a chamber of PRC 110M/GWP tubular furnace (Czylok, Jastrzębie-Zdrój, Poland). It is an advanced, digitally controlled furnace, consisting of a two–zoned tubular reactor. The first one is used to heat up incoming gas to the proper temperature, whereas the specimens are mounted in the second zone. Additionally, a steam generator is present. The furnace setup allows one to mix up to 5 different gases, due to 5 independent gas lines, controlled by dedicated electronic controllers. The oxidation process was conducted at 700 °C through 500 h with a steam flow of 200 g/h. For the mass measurements, the sample was cooled down with the furnace (~0.5 K/min) and heated up with the furnace (~8 K/min) again after weighting.

The mass of the sample was measured with MYA 5.4Y.B Plus microbalance of Radwag (Radom, Poland), characterized by a very high resolution (1 µg) and excellent standard deviation (s < 1 µg).

After the steam oxidation process, the sample was removed from the cooled down furnace, and its surfaces were examined using a scanning electron microscope (SEM-Merlin of Zeiss, Oberkochen, Germany). Furthermore, metallographic cross-sections were prepared. The samples were carefully cut with a low speed saw (Isomet 1000 of Buehler, Lake Bluff, IL, USA). On the surface of the sample, a thin layer of gold was evaporated to assure the electrical conductivity of the surface, which is needed to perform the process of Ni-electroplating. The latter allows one to protect the brittle oxide scale while further processing. Afterwards, the sample was cold embedded in LevoFast resin, ground and polished in a similar way to the surface of the coupons before the oxidation test. Since epoxy resin is an insulator, an approximately 5-nm-thick Au coating was sputtered on the surface of the cross section. Low voltage SEM-EDS allows one to obtain high quality and detailed maps of the selected elements [19].

Further investigations were performed with advanced transmission electron microscopy methods using Titan^3^ G2 60–300 (Thermo Fisher Scientific, Eindhoven, The Netherlands), equipped with a probe Cs corrector and ChemiSTEM system (Thermo Fisher Scientific, Eindhoven, The Netherlands). High resolution investigation in the scanning transmission mode (HRSTEM) supports the results of the selected area electron diffraction (SAED). JEMS software (Version 3.8431U2012, JEMS-SWISS, Jongny, Switzerland) was used to index the diffraction patterns and for high resolution image simulation [20]. In order to assure the highest accuracy, the diffraction mode of TEM was calibrated with the polycrystalline Ni sample. The chemical composition in the micro-areas was measured by energy-dispersive X-ray spectroscopy (EDS) as well as electron energy loss spectroscopy (EELS).

## 3. Results and Discussion

The results of the mass gain measurements up to 500 h are given in Table 2.

**Figure 1:**
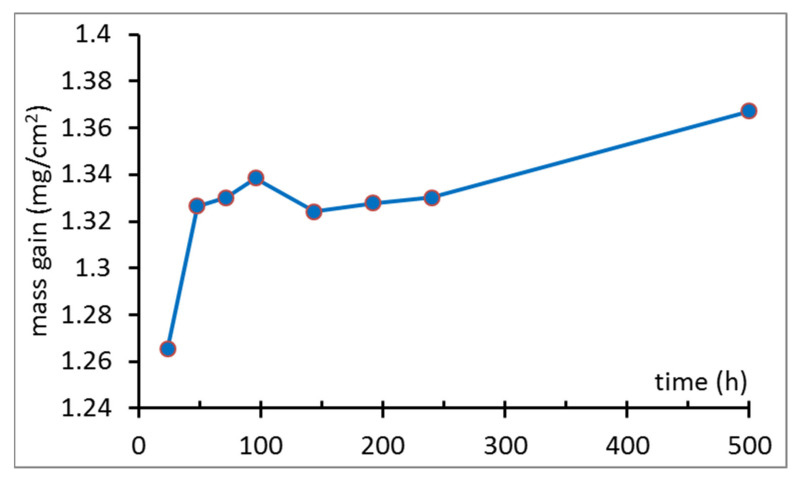
Mass gain after steam oxidation at 700 °C.

The highest mass gain is noticed after the first 24 h of oxidation, where the sample with a surface area of 344 mm^2^ bound ~4.36 mg of O (around 1.25 mg/cm^2^). After the next 24 h, another 211 μg was incorporated, and from this moment only slight changes were visible (13 and 28 μg after the next 24 and 48 h, respectively). The measurement after 144 h of oxidation clearly indicates spallation, since the measured value is lower than the one obtained previously; therefore, some amount of oxide was lost. The results of the mass gain measurements, normalized to the sample area, are visualized in Figure 1. Spallation while cooling down of the various materials was also reported in the literature [21,22].

**Figure 2:**
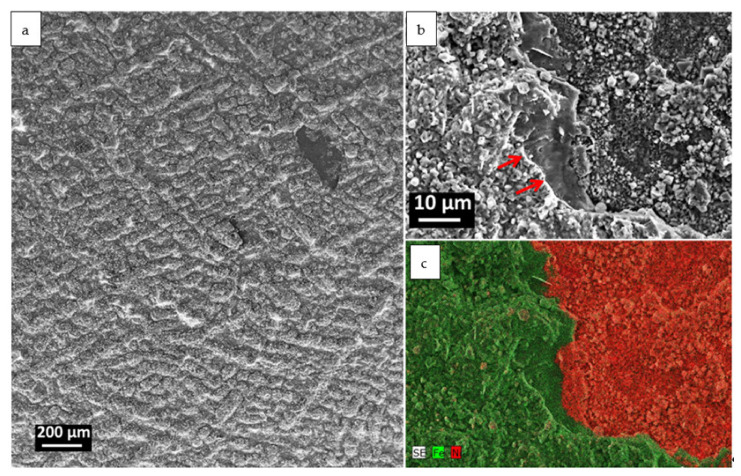
Surface of sample oxidized at 700 °C for 500 h in steam: (**a**) overview, secondary electron image (SEM-SE), (**b**) area damaged by spallation (SEM-SE), (**c**) elemental map of Fe and Ni.

The microstructure of the oxidized surface after 500 h of oxidation is shown in Figure 2a. Overall, the surface is homogenous, with a characteristic hill–valley microstructure, resembling dendritic arrays. However, some traces of spallation are visible too. Figure 2b shows the exemplary area damaged by spallation at higher magnification. The edge of the damaged area, indicated with a red arrow in Figure 2b is sharp, clear and unaffected with further corrosion, similar to the chips created at room temperature in ceramics, which suggests chipping while the cooling down process occurs. Such observations on ferritic steel were confirmed by Galerie et al. [21], where the highest stresses in the oxide scale occurred at around 170 °C during the cooling of Fe–18Cr–TiNb grade after oxidation at 750 °C. Moreover, at 800 °C, Fe_2_O_3_ has a thermal expansion coefficient of 12 ppm/K [23], whereas some Cr- and Fe-based spinels have a TEC of around 7 and 7–12 ppm/K, respectively [24]. In the current research, the surface of the oxide scale is composed of Fe oxide. Figure 2b shows that the area under the Fe oxide consists mainly of Ni-enriched (Figure 2c), fine-grained crystals, covered in some areas by the coarser grains.

**Figure 3:**
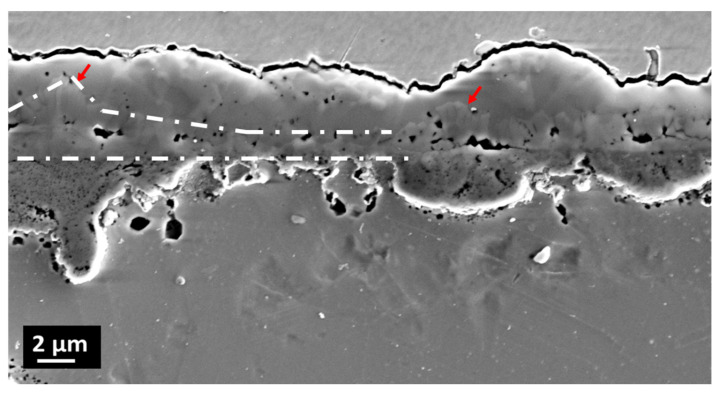
Cross section through oxide scale of the sample oxidized at 700 °C for 500 h in steam. The arrows indicate columnar crystals.

An image of the oxide scale cross section is visible in Figure 3. At first glance, based on the differences in the morphology of the layers, three of them can be distinguished (the boundaries indicated with white lines). The first one, right above the alloy surface is porous. The outermost layer is dense; however, some minor porosity is visible too. Between them, the intermediate layer is present. It contains columnar grown crystals (plates), marked with red arrows in Figure 3.

**Figure 4:**
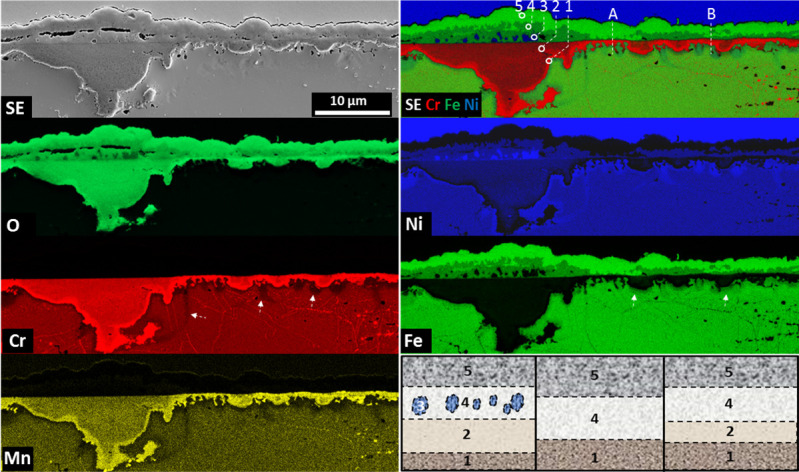
SEM-EDS analysis of the oxide scale after steam oxidation at 700 °C for 500h. (**a**) SE image of the investigated area, (**b**) compositional image of the selected elements, (**c**–**g**) elemental maps of the selected elements, (**h**–**j**) schematic drawing of the layers present in the oxide scale.

Low voltage SEM-EDS measurements, performed on the length of around 50 μm, reveal the complicated structure of the oxide scale and allow one to distinguish two additional layers (Figure 4).

According to Figure 4, a few layer combinations are possible. As an example, part of the oxide scale shown at the left side will be described. For the convenience of the description, each layer is numbered. Right above the alloy surface, a very thin, dense and continuous, Cr, Mn and O-containing layer is present, which would suggest the mixture of Cr and Mn oxides. Above it, in addition to the already mentioned elements, the presence of Ni was also detected. This layer is porous. Some crystals of the non-oxidized alloy (#3) are encapsulated between layer #2 and layer #4. The latter contains O, Ni and Fe. The outermost layer (#5) is built of Fe and O. It is worth mentioning that layer #4 consists of columnar grains (the arrows in Figure 3). The above described structure is schematically drawn in Figure 4h. Not all of the abovementioned layers are present along the surface of the oxidized sample. It is possible to have various combinations of them, such as the co-existence of layers #1, #4, #5 or #1, #2, #4, #5, marked in Figure 4b as “Area A” and “Area B”, respectively. A schematic diagram of the previously mentioned layouts is present in Figure 4i,j, respectively. Layers #1 and #2 are the effect of internal oxidation. Deep penetration in the material and the irregular interface between the base material and the internal oxidation zone are visible. The Cr-depleted areas and grain boundaries are well visible (marked with arrows in Figure 4e). Under the areas where layer #2 or #4 are forming, the Fe-depleted zone is present (the arrow in Figure 4f). The interface between the oxide scale and the internal oxidation zone is quite smooth.

Although the SEM-EDS investigation already gives plenty of information about the oxide scale, the determination of the phases present in the sample was performed by the method of scanning transmission electron microscopy (STEM), including high resolution imaging (HR) and selected area electron diffraction (SAED). It has to be underlined that TEM investigations are performed on relatively small areas in comparison to SEM; therefore, an oxide scale layer structure will be dependent on the area, from which lamella is extracted (see the composition and thickness variations in Figure 4b).

**Figure 5:**
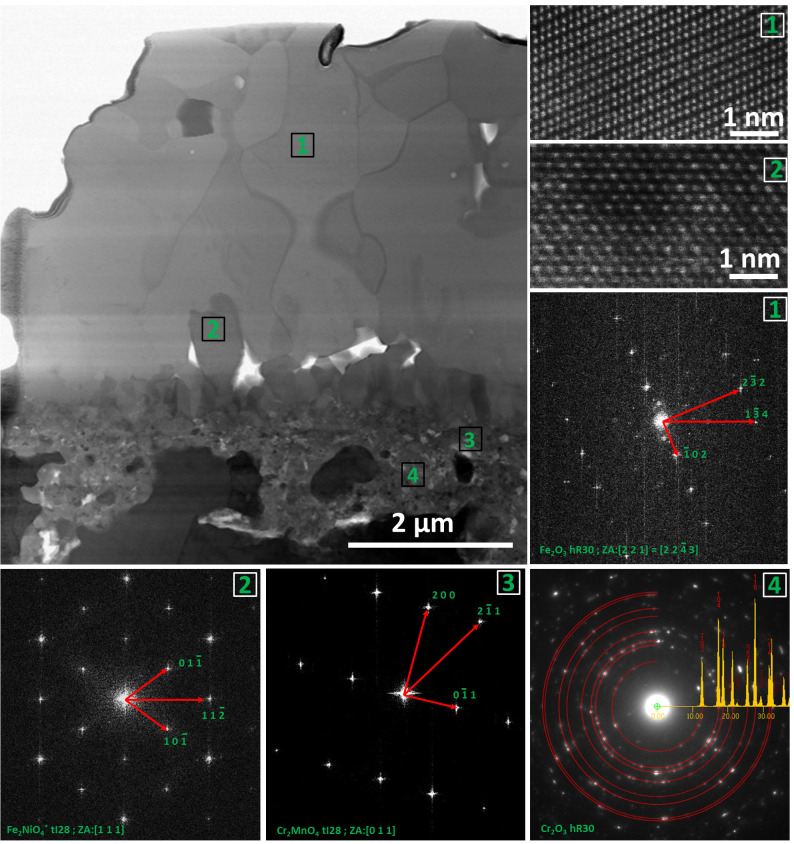
Scanning transmission electron microscopy (STEM) analysis of oxide scale after steam oxidation at 700 °C for 500h. (**a**) STEM-BF (bright field) image of the investigated area, (**b**) high resolution imaging (HR)STEM-HAADF (high-angle annular dark-field) image of area 1 shown in Figure 5a, (**c**) HRSTEM-HAADF image of area 2 shown in Figure 4a, (**d**–**f**) FFT from area 1, 2, 3, respectively (**g**) selected area electron diffraction (SAED) image of area 4.

Figure 5a shows the microstructure of the oxide scale on the cross-section. At first glance, three layers can be distinguished. The outermost (#5 in Figure 4) is the coarse crystalline one. This means there were a few crystals which started to grow rapidly. TEM-SAED and HRSTEM investigations allows the identification of this layer as Fe_2_O_3_ (hR30). Figure 4b shows the HR images of the area marked as 1 in Figure 4a, whereas in Figure 4e indexed fast Fourier transform (FFT) of Figure 4c is shown. Beneath it, a fine-grained layer is visible (#4 in Figure 4). Importantly, one plate which protrudes over the layer level is visible. It is in consistency with Figure 3, where the side surfaces of the plates are visible. Note the presence of pores at the Fe_2_O_3_/spinel interface. Such porosity might influence oxide scale integrity, due to the low contact area between the two phases, which in connection with stresses results in spallation. The HRSTEM-FFT analysis performed in area 2 (Figure 5a) revealed the tI28 spinel structure. Area 3 was identified as Cr_2_MnO_4_ tI28 spinel. The innermost layer is the effect of internal oxidation. The SAED investigations allow the identification of the layer as the polycrystalline Cr_2_O_3_ (hR30) phase. Please note also that some areas of the non-oxidized alloy are surrounded by oxides (#4 in Figure 3).

**Figure 6:**
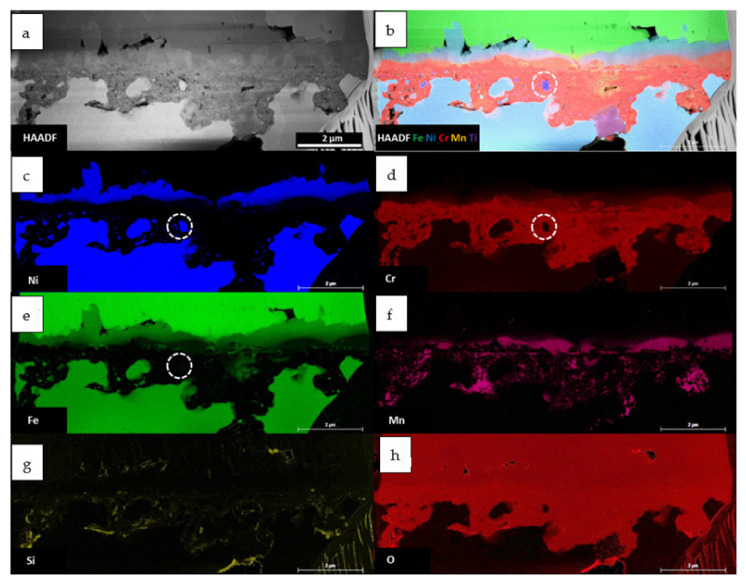
A STEM-EDS analysis of the oxide scale. (**a**) region of interest (STEM-HAADF), (**b**) compositional images of the selected elements, (**c**–**h**) elemental maps of the selected elements.

In Figure 6, the STEM-EDS results are visible. The outermost layer consists of Fe and O, which allows the confirmation of the Fe_2_O_3_ compound, as suggested by SAED. The innermost, internal oxidation zone consists mostly of Cr and O; however, Mn is also present in some areas. Some traces of SiO are also visible. It is worth mentioning that Ni oxidizing remains. The area indicated with a white circle in Figure 6b–e (the compositional image and the elemental maps of Ni, Cr and Fe, respectively) clearly shows that part of the non-oxidized bulk material consists of Ni only. Most interesting is the intermediate layer (#5 in Figure 4). The EDS results clearly show the presence of Fe and Ni in a 2:1 ratio, which in connection with the SAED results (Figure 4e) suggests the Fe_2_NiO_4_ tI28 spinel. Some difficulty was encountered in identification since there is no Fe_2_NiO_4_ tI28 spinel CIF database. Therefore, FFT from Figure 5e was indexed using the Cr_2_NiO_4_ database. The obtained data fit perfectly to the used standard; therefore, it was concluded that the discovered phase has a unit cell similar to that of tI28 Cr_2_NiO_4_; however, Fe is present instead of Cr. In order to confirm the absence of Cr in the test area, spectral imaging (SI) using an electron energy loss spectroscopy technique (EELS in STEM mode was performed.

**Figure 7:**
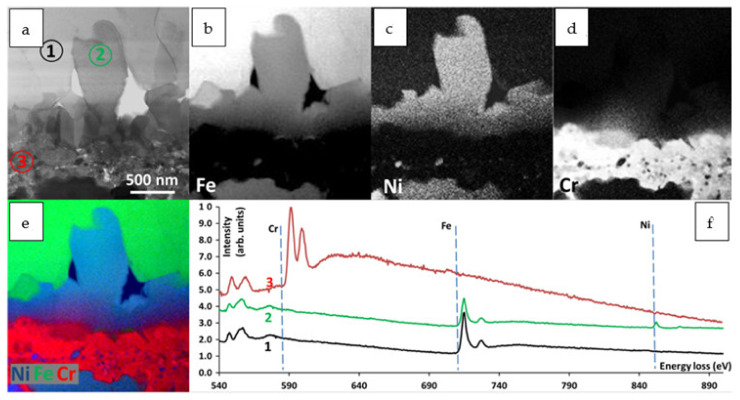
STEM-electron energy loss spectroscopy (EELS)-SI investigation: (**a**) region of interest (STEM-BF), (**b**–**d**) maps of the selected elements (EELS-SI), (**e**) compositional image of Fe, Ni and Cr, (**f**) EELS spectra for areas 1, 2, 3 (indicated in Figure 7a).

Figure 7a shows the region of interest; Figure 7b–d shows the elemental maps of Fe, Ni and Cr, respectively. A compositional image is shown in Figure 6e. The plot in Figure 7f shows the EELS spectra from areas 1, 2 and 3 (indicated Figure 7a). Spectrum 1 confirms the presence of O and Fe in the selected area. In area 2, in addition to Fe and O, the edge of Ni is also present; however, no traces of Cr are visible. A strong Cr edge is present in area 3. The performed investigations allow one to conclude that the upper part of the plate grown at the top of the layer #4 (Figure 4) represents the Fe_2_NiO_4_ tI28 spinel. Since the diffusion process is continuous at a high temperature and the Cr gradient is visible (Figure 6b and Figure 7d), it might be possible that Cr from the inner part of the oxide scale will partially replace Fe after a longer time of oxidation, or another spinel will be formed.

It is worth mentioning that the spallation, confirmed in Figure 2c, occurs between the coarse crystalline Fe_2_O_3_ and fine crystalline Fe_2_NiO_4_. The excessive formation of Fe_2_O_3_ at the top of the oxide scale is related to the grain size of the steel. The literature data confirm that coarse-grained 304 H steel exhibits a higher amount of Fe and a lower amount of Cr in the oxide scale in comparison to the fine-grained batch [25]. Moreover, the fine-grained (4 μm) TP347 steel develops a thin, protective Cr_2_O_3_ scale, whereas the coarse grain (65 μm) specimen developed a thick, two-layered oxide scale. The outermost layer was enriched in Fe, whereas Cr_2_O_3_ was grown on the steel surface [26]. Oxidation occurred much faster in the case of the coarse-grained sample. The smaller grain size resulted in a higher amount of grain boundary diffusion paths of Cr, which is in consistency with Figure 4b,e, where grain boundaries drained of Cr are visible. If the diffusion of Cr towards the surface is too slow (not enough diffusion paths), Fe will oxidize first. It could be concluded that the outermost layer is created due to the fast, outward diffusion of Fe. Cr is, due to an insufficient amount of diffusion paths, oxidized internally through the inward diffusion of O. The outward diffusion of Ni allows for a reaction with Fe_2_O_3_, resulting in Fe_2_NiO_4_ spinel creation. Since the grain size of the produced material is quite high, we expect to observe different oxide compositions grown on fine-grained Alloy 709.

## 4. Conclusions

1. The complex oxide scale was grown on the whole surface of the sample. Based on the morphology and chemical composition of its constituents, it can be divided into a few layers.
2. The outermost part of the oxide scale is built of a thick layer of coarse-grained Fe_2_O_3_. Excessive formation of the hematite is caused by the coarse grain (~52 μm) of the alloy, which reduces the Cr diffusion rate. The hematite layer has a spallation tendency while cooling.
3. Beneath the hematite layer, the presence of Fe_2_NiO_4_ spinel was detected. It has a tetragonal (tI28) structure. Increased porosity is visible at the interface between hematite and Fe_2_NiO_4_. Cr underwent an internal oxidation process.
4. The SEM-EDS investigation performed under lowered voltage is a suitable method to obtain valuable information concerning the chemical composition of even very thin oxide layers. It allows one to obtain high quality elemental distribution maps. For the full characterization of present phases, TEM/STEM investigations are necessary. STEM-EELS is an adequate technique to investigate oxide scales.

## Figures and Tables

**Table 1:** The average chemical composition of the investigated steel, measured with optical emission spectrometry.

Element	Fe	C	Ni	Cr	Mo	Mn	Si	Nb	Ti	P	S
Wt.%	balance	0.18	24.73	20.30	1.31	0.91	0.41	0.24	0.11	0.01	0.01
Std. Dev.	–	0.01	0.15	0.17	0.01	0.02	0.01	0.01	0.00	0.00	0.00

**Table 2:** Mass gain after steam oxidation at 700 °C.

Parameter	t_0_	t_1_	t_2_	t_3_	t_4_	t_5_	t_6_	t_7_	t_8_
time (h)	0	24	48	72	96	144	192	240	500
mass (mg)	2088.764	2093.120	2093.331	2093.344	2093.373	2093.323	2093.336	2093.344	2093.472
mass to area (mg/cm^2^)	606.6255	607.8907	607.9519	607.9557	607.9639	607.9496	607.9534	607.9557	607.9928
mass gain (t_n_−t_0_)	0.000	1.265	1.326	1.330	1.338	1.324	1.328	1.330	1.367
mass gain (t_n_−t_n−1_)	0.000	1.265	0.061	0.004	0.008	-0.014	0.004	0.002	0.037

## Data Availability

The raw/processed data required to reproduce these findings cannot be shared at this time as the data also form part of an ongoing study.
